# Metabolic phenotype mediates the outcome of competitive interactions in a response‐surface field experiment

**DOI:** 10.1002/ece3.8388

**Published:** 2021-12-10

**Authors:** Lukas Schuster, Craig R. White, Dustin J. Marshall

**Affiliations:** ^1^ Centre for Geometric Biology School of Biological Sciences Monash University Melbourne Vic. Australia

**Keywords:** intraspecific competition, life history, metabolic rate, reproductive output, trait variation

## Abstract

Competition and metabolism should be linked. Intraspecific variation in metabolic rates and, hence, resource demands covary with competitive ability. The effects of metabolism on conspecific interactions, however, have mostly been studied under laboratory conditions. We used a trait‐specific response‐surface design to test for the effects of metabolism on pairwise interactions of the marine colonial invertebrate, *Bugula neritina* in the field. Specifically, we compared the performance (survival, growth, and reproduction) of focal individuals, both in the presence and absence of a neighbor colony, both of which had their metabolic phenotype characterized. Survival of focal colonies depended on the metabolic phenotype of the neighboring individual, and on the combination of both the focal and neighbor colony metabolic phenotypes that were present. Surprisingly, we found pervasive effects of neighbor metabolic phenotypes on focal colony growth and reproduction, although the sign and strength of these effects showed strong microenvironmental variability. Overall, we find that the metabolic phenotype changes the strength of competitive interactions, but these effects are highly contingent on local conditions. We suggest future studies explore how variation in metabolic rate affects organisms beyond the focal organism alone, particularly under field conditions.

## Graphical abstract

We provide one of the first formal studies of metabolic rate effects on intraspecific competition under field conditions. By using a response‐surface design, we show that the metabolic phenotypes of both the focal individual and the competitor change the strength of competitive interactions, but these effects are highly contingent on local conditions.

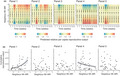

## 1 INTRODUCTION

An individual's metabolic rate and competitive ability are tightly linked (Biro & Stamps, 2010; Sloman & Armstrong, 2002). Competition for resources among and within species constrains the acquisition of energy by individuals and, consequently, their growth and fitness. Similarly, an individual's metabolic rate is tightly linked to its resource demands, with individuals with higher metabolic rates having greater resource requirements (Brown et al., 2004; Burton et al., 2011). At high population densities especially, competition limits *per capita* resource availability (exploitative competition) or access to resources (interference competition) (Antonovics & Levin, 1980; Violle et al., 2010). Accordingly, individuals sometimes downregulate their metabolic rates in response to intraspecific competition so as to maintain positive energy fluxes despite lower resource availability (DeLong et al., 2014; Ghedini et al., 2017; but see Yashchenko et al., 2016). Yet competitive environments do not always favor lower, “more thrifty” metabolic phenotypes.

Higher metabolic rate individuals can increase their competitive ability by increasing access to, or use of, scarce resources due to their higher activity levels, greater boldness, territorial aggression, and competitive dominance (Biro & Stamps, 2010; Careau et al., 2008). Similarly, metabolic rate is known to covary with a range of traits that can influence resource acquisition—individuals with higher metabolic rates may forage more voraciously or effectively (Biro & Stamps, 2010; Chappell et al., 2007; McNab, 1980). Consequently, individuals with higher metabolic rates may be able to gain preferential access to resources or extract disproportionately more resources from the environment (Pettersen et al., 2020), potentially resulting in an asymmetric competition between metabolic phenotypes (Weiner, 1990). To date, competitive interactions among conspecifics of varying metabolic phenotypes have mostly been studied under laboratory conditions where conditions are more constant than conditions in the real world (Ward et al., 2006). Consequently, field studies investigating how metabolic rate affects competition under natural, more variable conditions are a necessary next step (Álvarez & Nicieza, 2005).

The competitive advantages conferred by any one metabolic phenotype should depend strongly on environmental conditions (Killen et al., 2013). A higher metabolic rate is often associated with a higher energy turnover that can be beneficial for growth and survival when resource availability is high but disadvantageous when resource levels are low (Armstrong et al., 2011; Auer et al., 2015, 2020; Bochdansky et al., 2005; Burton et al., 2011; Killen et al., 2011). Individual differences in metabolic rate and associated traits such as body size may therefore interact with environmental conditions to determine the outcome of competitive interactions among conspecifics. For example, individuals with higher metabolic rates may only be able to grow larger and, therefore, be competitively dominant if the *per capita* resource levels are high (Ward et al., 2006). Conversely, higher metabolic rates may be disadvantageous in resource‐limited environments if resources are simply insufficient to sustain individuals with higher energy demands (Auer et al., 2020). How environmental conditions interact with metabolic rate to determine competitive outcomes in the field, however, remains poorly understood.

In a manipulative field experiment, we examined how metabolic rate mediated conspecific interactions in the bryozoan, *Bugula neritina*, a colonial, sessile marine invertebrate. Colonies of *B*. *neritina* are commonly found as part of fouling communities throughout the world where they form dense congregations of conspecifics, which may result in intense competitive interactions among individuals. We took advantage of the natural and persistent variation in metabolic rate among individual *B*. *neritina* colonies (Pettersen et al., 2016, 2020; Schuster et al., 2019, 2021) to test for the effects of metabolic rate on competition. We used a trait‐specific response‐surface design to create pairwise interactions of individuals with differing metabolic rates (Cameron et al., 2019; Inouye, 2001). Due to the sessile nature of *B*. *neritina*, we were able to follow the performance of individuals across their entire lives in the field. We then measured the outcomes of pairwise interactions by comparing the performance (survival, growth, and lifetime reproductive output) of individuals with different metabolic rates, both in the presence and absence of a neighbor colony.

## 2 MATERIALS AND METHODS

### 2.1 Study species, site, and field deployment

*Bugula neritina* Linnaeus, 1758, is an arborescent bryozoan common to sessile marine communities worldwide. *B*. *neritina* grows by asexual budding of connected zooids (individual subunits) at the distal end to produce symmetrical branching colonies (Keough, 1989; Keough & Chernoff, 1987). Once sexually mature, colonies form clearly visible structures called ovicells (Woollacott & Zimmer, 1975). Each ovicell broods a single larva, which is released into the plankton once embryogenesis is complete. Upon release, the nonfeeding larvae are immediately competent to settle, and most larvae settle within hours under field conditions (Burgess & Marshall, 2011). Larvae also preferentially settle close to conspecifics in the laboratory (Keough, 1984), and such aggregations of *B*. *neritina* conspecifics are often observed in the field.

We collected sexually mature *B*. *neritina* colonies in Port Phillip Bay, Victoria, Australia (37°51’43.3”S, 144°57’51”E) in April 2019. To obtain individuals for our experiments, we spawned colonies according to standard procedures (Schuster et al., 2019). Briefly, we kept colonies in the laboratory in field‐collected seawater in aerated tanks in the dark. After 48 h, we spawned colonies by exposing them to bright light and settled single larvae in a drop of seawater on roughened A4 acetate sheets to induce settlement (~150 settlers per acetate sheet). After 3 h, we rinsed unsettled larvae from the acetate sheets and kept settlers in tanks with unfiltered seawater. The next day, we attached the A4 acetate sheets bearing settlers to five PVC backing panels (57 × 57 × 0.6 cm; two acetate sheets per panel) and suspended the panels 1 m below the water surface with settlers facing down at the Royal Brighton Yacht Club (37°54′25″S, 144°58′54″E).

### 2.2 Mass‐independent metabolic rate

To conduct metabolic rate measurements, we returned acetate sheets bearing settlers to the laboratory after they had been in the field for 2 weeks. We kept colonies in aerated tanks with field‐collected seawater at 19°C overnight. Prior to metabolic rate measurements, we removed any epibionts and debris from the colonies. We then separated individual colonies from the A4 sheets by cutting around the base of the colonies such that each colony was attached to a small square of acetate sheet. In total, we measured the metabolic rates of 372 colonies using 750‐µl glass vials (Loligo Systems, Denmark) and 24‐channel PreSens sensor dish readers (SDR2, PreSens, Germany). We determined metabolic rates as oxygen consumption rates at 19°C over 3 h as described in Schuster et al. (2019). We then converted estimates of V̇O_2_ (ml h^−1^; White et al., 2011) to metabolic rates (mJ h^−1^) using the calorific conversion factor of 20.08 J ml^−1^ O_2_ (Crisp, 1971).

We estimated size‐independent metabolic rates (MI‐MR) by regressing metabolic rate on colony size (nonlinear regression of the form $MR=a*M^{b}$, where *MR* is metabolic rate, *M* is colony size, *a* is the intercept, and *b* is the scaling exponent) and extracting the residuals. To determine colony size, we counted the number of zooids in each colony. Given that colonies were attached to squares of acetate sheet, zooid counts were more reliable than weighing them, and the number of zooids and colony mass are strongly correlated (Schuster et al., 2019). Colonies used for metabolic rate measurements ranged from 16 to 48 zooids in size.

### 2.3 Experimental design and field deployment

**FIGURE 1:**
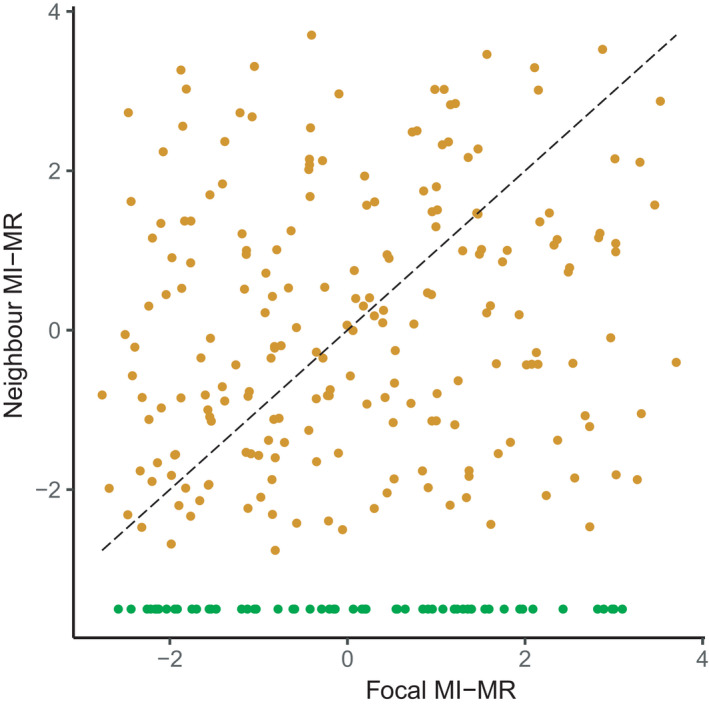
Schematic of the trait‐specific, response‐surface design used to test the effects of metabolism on pairwise interactions between *Bugula neritina* colonies. The orange points show the combinations of focal and neighbor colony mass‐independent metabolic rates (MI‐MR) used in pairwise interactions (*n* = 206); the green points show the mass‐independent metabolic rates of colonies grown without a neighbor colony (*n* = 59). The gray‐dashed line indicates equivalences between focal and neighbor MI‐MRs

Our main goal was to investigate whether metabolic rate mediates the outcome of pairwise interactions using a trait‐specific, response‐surface design (Cameron et al., 2019; Inouye, 2001). Based on the continuous range of metabolic rates from our source population (1.25–7.67 mJ h^−1^ *absolute* metabolic rates), we generated pairwise combinations of metabolic rates (maximum difference in MI‐MR between pairs: 5.19; maximum *absolute* differences in metabolism between pairs: 5.08 mJ h^−1^; Figure 1). To create our treatments, we glued two acetate sheet squares, each bearing a single colony, onto PVC plates (55 × 55 × 3 mm) such that colonies grew at a distance of 1 cm from each other. We treated both these colonies as the focal colony and neighbor colony to test for reciprocal interactions (Inouye, 2001). In addition, we estimated the baseline relationship between metabolic rate and performance of single colonies without a neighbor colony by gluing a blank acetate sheet square 1 cm from a focal colony (distance between the center of the empty acetate square and the focal colony). We then distributed a total of 162 plates across the five backing panels and redeployed them into the field. Within each panel, we attached plates at a distance of at least 5 cm from each other in order to minimize competitive interactions with colonies on neighboring plates. It is noteworthy that we assigned colonies haphazardly to each panel. Consequently, there were no differences in focal colony size or MI‐MR between panels (mean ± SE; Panel 1: zooids: 29.1 ± 6.9, MI‐MR: 0.08 ± 0.23; Panel 2: zooids: 29.15 ± 0.68, MI‐MR: 0.09 ± 0.22; Panel 3: zooids: 29.82 ± 0.83, MI‐MR: 0.14 ± 0.25; Panel 4: zooids: 29.91 ± 0.56, MI‐MR: −0.03 ± 0.2; Panel 5: zooids: 28.89 ± 0.69, MI‐MR: 0.1 ± 0.23; one‐way ANOVA: zooids: *F*_4,260_ = 0.44, *p* = .78, MI‐MR: *F*_4,260_ = 0.08, *p* = .99).

We followed the performance of 265 colonies of known metabolic rates throughout their entire life history, until all colonies had died (April through to October 2019). We followed the survival, growth, and reproductive output of each colony every 2 weeks. Colonies were considered alive if they were still attached to the plate and >10% of the colony contained feeding zooids. We measured the reproductive output of each colony by counting the number of ovicells throughout the duration of the experiment, and growth as the number of bifurcations at each measurement point (Keough & Chernoff, 1987). We also removed any nonexperimental settlers (both *Bugula* and other species) from the plate at each measurement point to eliminate competition from other organisms. Furthermore, to avoid any environmental effects and effects from neighboring colonies on other plates associated with a focal colony's position within a panel (i.e., in the center surrounded by lots of competitors vs. at the edge) on metabolic rates and performance, we moved each plate to a different position within the assigned panel every 2 weeks (Mitchell‐Olds & Shaw, 1987; Rausher, 1992).

### 2.4 Statistical analyses

We conducted two different sets of analyses using generalized linear models (GLMs) and repeated measures analyses of covariance (RM ANCOVA): we tested (i) the effects of neighbor colony presence (denoted “1”) or absence (i.e., colonies grown in isolation; denoted “0”), and (ii) the effects of neighbor MI‐MR and focal MI‐MR on focal colony survival at 20 weeks (c.f. Pettersen et al., 2016, 2020), growth, the *per capita* reproductive output over time, and the cumulative reproductive output after 24 weeks (i.e., an individual's summed reproductive output across the life history), respectively. For survival, we conducted a binomial GLM with a logit‐function, with focal MI‐MR (continuous fixed effect), panel ID (categorical fixed effect), and either neighbor colony presence/absence (categorical fixed effect; “1” or “0”) or neighbor MI‐MR (continuous fixed effect) included in the model. For the cumulative reproductive output, we conducted a quasi‐Poisson GLM with a log‐link function using the same model structure as above. For growth and the *per capita* reproductive output over time, we conducted RM ANCOVAs with focal MI‐MR (continuous fixed effect), panel ID (categorical fixed effect) and time (measurement points; categorical fixed effect), and either neighbor colony presence/absence (categorical fixed effect; denoted “1” or “0”) or neighbor MI‐MR (continuous fixed effect) included in the model. For growth analyses, we also included initial colony size as a fixed effect in analyses to account for differences in focal colony sizes at the start of the experiment. As the response variable, we used either size (number of zooids; log_10_‐transformed prior to analyses) or the *per capita* reproductive output (log_10_‐transformed prior to analyses) of focal colonies at each measurement point, respectively. To derive colony size in terms of number of zooids within a colony (for colonies >2 weeks of age), we converted the number of bifurcations to zooid number by assuming an average of four pairs of zooids in between branching points of a colony (Keough & Chernoff, 1987).

For all analyses, we first fit full models and reduced these where appropriate by removing nonsignificant interactions (assessed from log‐likelihood ratio tests for binomial GLMs or *F*‐ratio tests for Gaussian RM ANCOVAs and quasi‐Poisson GLMs; where *α* > 0.05). For focal colony survival and growth, we found significant three‐way interactions (survival: panel × focal MI‐MR × neighbor colony presence/absence and panel × focal MI‐MR × neighbor MI‐MR; growth: panel × neighbor MI‐MR × time), which were driven by one panel (see Results). We, therefore, performed additional analyses but excluded this panel to test for main effects and their interactions on focal colony survival and growth on the other four panels. We performed pairwise *t*‐tests to compare survival and cumulative reproductive outputs between focal colonies that grew in the presence of a neighbor and focal colonies that grew in the absence of a neighbor on each panel. We conducted all statistical analyses in R version 4.1 (R Core Team, 2017) using the packages *lme4* (Bates et al., 2015) and *car* (Fox & Weisberg, 2019).

## 3 RESULTS

### 3.1 The effect of neighbor colony presence/absence on focal colony performance

**FIGURE 2:**
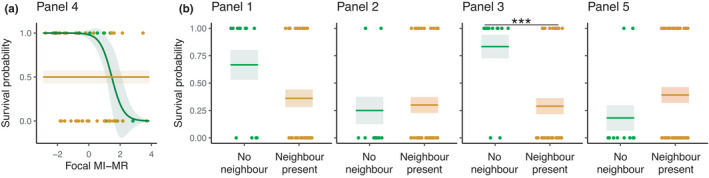
The effects of (a) neighbor colony presence/absence and focal MI‐MR and (b) neighbor colony presence/absence on focal colony survival probability on each panel. Green and orange dots show underlying data points for focal colonies grown in the absence or presence of a neighbor colony, respectively; lines show predicted survival probability from generalized linear models (± 95% CI)

After 20 weeks in the field, we found that focal colony survival depended on the interaction between neighbor colony presence/absence and focal MI‐MR, but the nature of this interaction varied among panels (panel × neighbor colony presence/absence × focal MI‐MR: *χ* ^2^ = 6.8, *df* = 1, *p* = .009). On one panel, focal colonies grown in the absence of a neighbor colony survived better if they had a lower metabolic rate, but focal MI‐MR did not affect survival if a neighbor colony was present (Figure 2a; Panel 4). Focal MI‐MR did not affect focal colony survival on the other four panels (Panels 1–3 and 5; panel × focal MI‐MR: *χ* ^2^ = 2.69, *df* = 1, *p* = .1; focal MI‐MR: *χ* ^2^ = 0.84, *df* = 1, *p* = .36; Figure 2b). Instead, we detected a significant panel ×neighbor colony presence/absence interaction effect on focal colony survival on these panels (*χ* ^2^ = 13.44, *df* = 1, *p* = .0002), whereby neighbor colony presence decreased focal colony survival on one panel (Panel 3).

**FIGURE 3:**
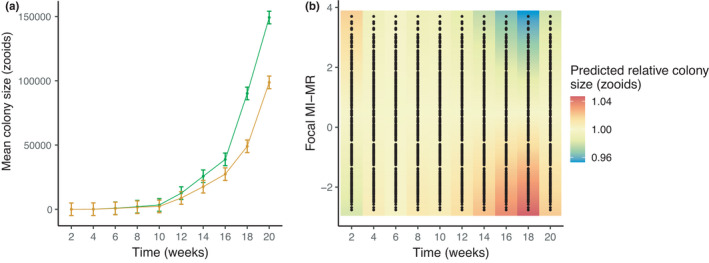
(a) Mean colony size (as number of zooids) of focal colonies grown in the absence (green line) or presence of a neighbor colony (orange line) plotted against time (in weeks). (b) The effect of focal MI‐MR on relative colony size (as the number of zooids; standardized to the mean) over time (in weeks). Error bars in (a) indicate standard errors. Black dots in (b) show underlying data points; warmer colors depict larger relative colony sizes

The presence of a neighbor colony invariably reduced focal colony growth, with focal colonies being on average 33.8% smaller in terms of zooid number after 20 weeks in the field compared to focal colonies grown in the absence of a neighbor colony (time × neighbor colony presence/absence: *F*_9,1966_ = 2.83, *p* = .003; Figure 3a). Focal colonies with lower metabolic rates grew larger than higher metabolic rate individuals (time × focal MI‐MR: *F*_9,1966_ = 3.08, *p* = .001; Figure 3b), regardless of whether a neighbor colony was present (time × neighbor colony presence/absence × focal MI‐MR: *F*_9,1966_ = 0.82, *p* = .59).

**FIGURE 4:**
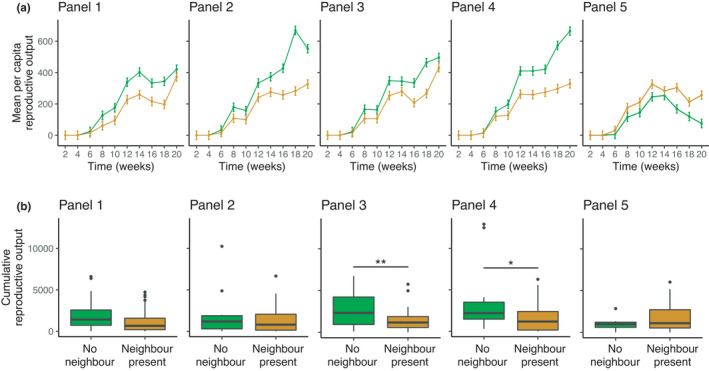
The effect of neighbor colony presence/absence on (a) the mean *per capita* reproductive output of focal colonies over time (± 1 SE); and (b) the cumulative reproductive outputs of focal colonies on each panel. Lines in (a) show mean *per capita* reproductive outputs of colonies grown in the absence (green) or presence of a neighbor (orange). Boxplots in (b) show the distribution of cumulative reproductive outputs for colonies grown in isolation (green) or in the presence of a neighbor (orange)

In terms of *per capita* reproductive output, we found that the effect of neighbor colony presence/absence differed across panels and over time (panel ×neighbor colony presence/absence × time: *F*_36,1975_ = 1.42, *p* = .05). The interaction was driven by one panel, on which focal colonies had relatively higher *per capita* reproductive outputs if a neighbor colony was present (Figure 4a; Panel 5). On the other four panels, colonies grown without a neighbor colony had overall higher *per capita* reproductive outputs (Figure 4a; Panels 1–4). Focal MI‐MR did not affect *per capita* reproductive outputs of focal colonies (focal MI‐MR × time: *F*_9,1975_ = 0.43, *p* = .92), regardless of whether another colony was present or absent (focal MI‐MR × neighbor colony presence/absence × time: *F*_9,1975_ = 1.45, *p* = .16).

In terms of cumulative reproductive outputs of focal colonies (i.e., the summed reproductive outputs across each census date), the effect of neighbor colony presence/absence varied among panels (panel ×neighbor colony presence/absence: *F*_4,254_ = 2.25, *p* = .06). On two panels, colonies grown in the absence of a neighbor colony produced on average 46% more offspring than colonies that were grown in the presence of a neighbor (Figure 4b). Focal MI‐MR did not affect cumulative reproductive outputs of focal colonies (*F*_1,254_ = 2.54, *p* = .11), although focal colonies with lower metabolic rates tended to have higher reproductive outputs.

Our results pertaining to the effects of neighbor colony presence/absence are summarized in Tables 1 and 2.

**TABLE 1:** Summary of survival, growth, and reproductive outputs, and the various effects of neighbor colony presence/absence across all experimental panels. The significance levels of neighbor colony presence/absence effects within each panel are presented in Table 2

Performance metric	Panel 1	Panel 2	Panel 3	Panel 4	Panel 5
Survival					
Neighbor colony presence					
Neighbor colony presence × focal MI‐MR					
Growth					
Neighbor colony presence					
Time × focal MI‐MR					
Time × neighbor colony presence					
*Per capita* reproductive outputs					
Time × neighbor colony presence					
Cumulative reproductive outputs					
Neighbor colony presence					

Note

Purple indicates the response variable increased with neighbor colony presence, red indicates the response variable decreased with neighbor colony presence and with higher focal metabolic rate.

**TABLE 2:** Outcome of pairwise *t*‐tests comparing the survival or cumulative reproductive outputs between focal colonies that were grown in the presence and focal colonies that were grown in the absence of a neighbor colony on each panel

	Neighbor absent		Neighbor present		*p*
	Mean	SE	Mean	SE	
Survival					
Panel 1	0.67	0.14	0.36	0.08	.07
Panel 2	0.25	0.13	0.3	0.07	.74
Panel 3	0.83	0.11	0.29	0.07	**.0006**
Panel 5	0.18	0.12	0.39	0.07	.2
Cumulative reproductive outputs					
Panel 1	2,202.08	2,363.78	1,276.22	238.66	.11
Panel 2	2,009.08	842.51	1,358.18	243.64	.31
Panel 3	2,795.67	667.78	1,402.11	197.74	**.009**
Panel 4	3,744.75	1,250.82	1,615.39	254.73	**.01**
Panel 5	1,004.73	224.93	1,696.83	225.91	.15

Note

Bolded values are significant at α < 0.05.

### 3.2 The effect of neighbor MI‐MR on focal colony performance

**FIGURE 5:**
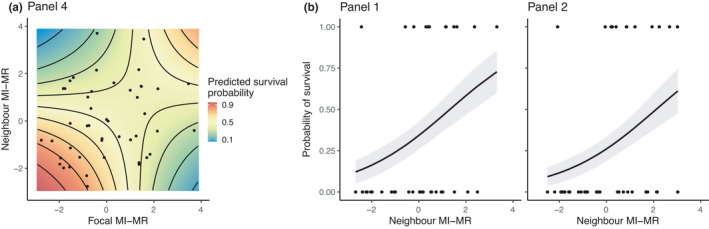
The effect of (a) focal MI‐MR and neighbor MI‐MR and (b) neighbor MI‐MR on focal colony survival probability on panels 1, 2, and 4. Black dots show underlying data points. (a) Warmer colors depict a higher predicted survival probability. Lines in (b) show predicted survival probability from generalized linear models (±95% CI)

Survival of focal colonies depended on the metabolic rate of both the focal colony and the neighboring colony, and these effects varied among panels (panel × focal MI‐MR ×neighbor MI‐MR: *χ* ^2^ = 5.33, *df* = 1, *p* = .02). On one panel, we found that focal colonies with a lower metabolic rate survived better if they were paired with a low metabolic rate neighbor colony (Figure 5a; Panel 4). On the other panels, neighbor metabolic rate affected focal colony survival on some panels but not on others (panel ×neighbor MI‐MR: *χ* ^2^ = 7.29, *df* = 1, *p* = .007). On two panels, we found a positive relationship between neighbor MI‐MR and focal colony survival (Figure 5b; Panels 1 and 2), but on the other two panels there was no effect of neighbor MI‐MR (Panels 3 and 5; *χ* ^2^ = 0.23, *df* = 1, *p* = .63).

**FIGURE 6:**
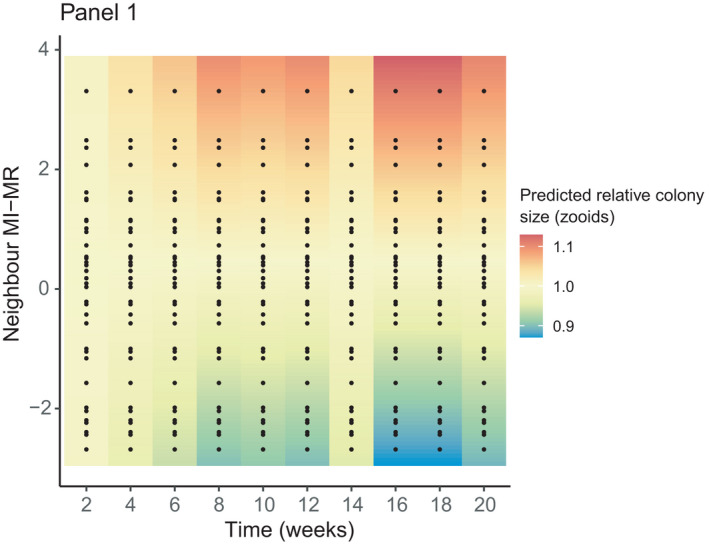
The effect of neighbor MI‐MR on the predicted relative colony size (in zooids; standardized to the mean) over time (in weeks). Black dots show underlying data points. Warmer colors depict higher relative colony sizes

Neighbor metabolic rate affected the growth of focal colonies, but, again, the effect varied across panels (panel × neighbor MI‐MR × time: *F*_36,1471_ = 1.45, *p* = .04). On one panel, focal colonies grew larger if they were paired with a high metabolic rate neighbor (Figure 6; Panel 1), whereas neighbor MI‐MR did not affect focal colony growth on the other panels (Panels 2–5; panel × neighbor MI‐MR × time: *F*_27,1222_ = 0.86, *p* = .68).

**FIGURE 7:**
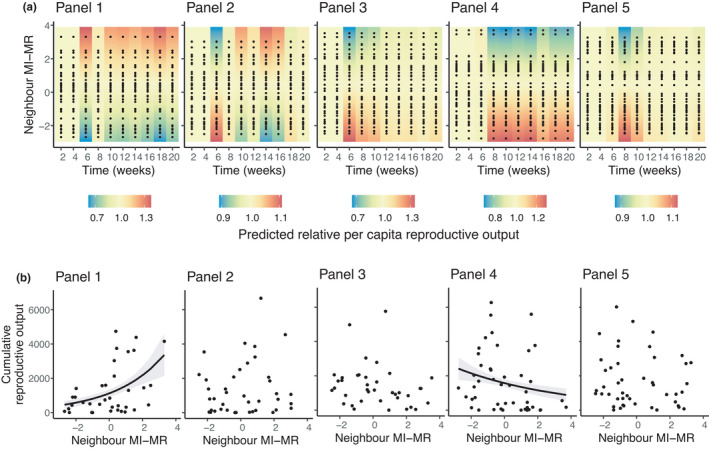
The effect of neighbor MI‐MR on (a) the relative *per capita* reproductive output (standardized to the mean) over time (in weeks); and (b) the cumulative reproductive outputs of focal colonies on each panel. Black dots in (a) and (b) show underlying data points. Warmer colors in (a) depict higher relative *per capita* reproductive outputs. Lines in (b) are the predicted lines of best fit from generalized linear models (±95% CI)

We detected a significant effect of neighbor MI‐MR on the *per capita* reproductive output of focal colonies, but the effect differed across panels (panel × neighbor MI‐MR × time: *F*_36,1507_ = 1.53, *p* = .02). Overall, the neighbor MI‐MR effect was strongest when reproduction began (Figure 7a)—focal colonies paired with a low metabolic rate neighbor colony had relatively higher reproductive outputs on all except one panel (Panel 1), where the effect was reversed. After 8 weeks, the effect of neighbor MI‐MR on focal colony *per capita* reproductive outputs persisted over time on two panels (Panels 1 and 4) but dissipated on two other panels (Panels 3 and 5). On one panel (Panel 2), focal colonies paired with a low metabolic rate neighbor colony reproduced more during early stages, but the effect changed in sign at 10 weeks and focal colonies paired with a high metabolic rate neighbor reproduced more thereafter.

Neighbor MI‐MR also affected the cumulative reproductive output of focal colonies, but, again, the effect differed across panels (panel ×neighbor MI‐MR: *F*_4,195_ = 2.9, *p* = .02). Here, on two panels, we found either a positive (Panel 1) or negative relationship (Panel 4) between neighbor MI‐MR and cumulative reproductive outputs of focal colonies (Figure 7b). On the other three panels, we could not detect an effect of neighbor MI‐MR (panel × neighbor MI‐MR: *F*_1,119_ = 0.24, *p* = .62) on the cumulative reproductive output of focal colonies (Figure 7b; Panels 2, 3 and 5). These effects on cumulative reproductive outputs mostly reflect our results for biweekly *per capita* reproductive rates—when effects persisted through time, they were reflected in cumulative reproductive outputs.

Our results pertaining to the effects of neighbor metabolic rate are summarized in Table 3.

**TABLE 3:**
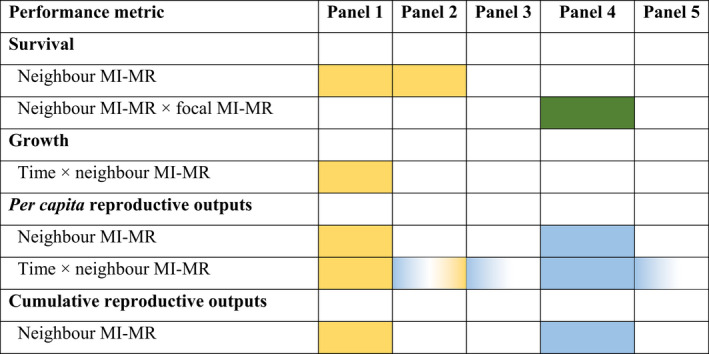
Summary of survival, growth, and reproductive outputs, and the various effects of neighbor metabolic rate across all experimental panel

*Note:* Yellow indicates the response variable increased with neighbor metabolic rate; blue indicates the response variable decreased with neighbor metabolic rate. Green indicates an interaction between both neighbor and focal metabolic rate. Color gradients indicate a change in sign of the effect over time, for example, a change from a negative to a positive effect (blue to yellow) or a change from a negative to no effect (blue to white). We used simple main‐effects tests to determine the significance of main effects (and their interactions) within each panel.

## 4 DISCUSSION

We found that the metabolic phenotype of conspecific neighbors altered the performance of focal colonies, but these effects showed strong microenvironmental variability. On most panels, we observed competitive effects—the presence of a neighbor reduced the performance of focal colonies. The effects of metabolic phenotype (of both the focal individuals and their neighbors), however, were complex and pervasive, affecting survival, growth, and lifetime reproductive outputs. Here, the strength and even the sign of metabolic effects varied among panels and potentially with local resource regimes. Overall, these findings suggest a strong context dependence of metabolic rate effects on conspecific interactions of *B*. *neritina*. We found significant metabolic rate effects on a range of performance metrics (i.e., survival, growth, *per capita*, and cumulative reproductive outputs) of focal colonies—but we had strong statistical evidence for these effects varying among panels. While such results are less straightforward, our findings highlight the importance of exploring the effects of metabolism under realistic conditions with replication in space. Had we not replicated in space, we would have concluded that the effects of neighbor metabolism are more consistent than they actually are. This small spatial variation in the effects of metabolism on performance is likely to maintain variance in this trait (Lange et al., 2016).

We found that individuals with higher metabolic rates grew less than individuals with lower metabolic rates. Recent studies have shown that individuals with lower metabolic rates often grow more and reach larger body sizes due to their relatively lower maintenance costs (Burton et al., 2011; Pettersen et al., 2018), particularly when *per capita* resource availabilities are scarce (Auer et al., 2015, 2020; Reid et al., 2011, 2012; Zeng et al., 2017; Zeng, Zhang, et al., 2017). Thus, within a population where conspecifics compete for resources, a lower metabolic rate may confer a growth advantage when resources are limiting.

Colonies growing in the presence of neighbors with lower metabolic rates, which grew to larger colony sizes, tended to grow and reproduce more themselves on some panels. This benefit may have arisen for one of two reasons: (1) neighbors with lower metabolic rates fed less voraciously, leaving more food for the focal colonies (this seems unlikely given these neighbors were larger overall, and so have higher total resource consumption rates); or (2) slower metabolism and, therefore, larger neighboring colonies, may have altered local flow regimes to benefit focal colonies more. Previous studies in this system indicate that conspecific size is a key mediator of the delivery of resources to interacting individuals (Cameron & Marshall, 2019; Cameron et al., 2017), and we suspect size, rather than *per capita* resource consumption drives our results here. In aquatic systems (including our own), the physical structure of sessile organisms can disrupt boundary currents and increase resource entrainment, particularly when water currents are too fast (Cameron et al., 2019; Cameron & Marshall, 2019; Okamura, 1984; Svanfeldt et al., 2017). Thus, it is possible that focal colonies likely benefited from being adjacent to fast growing, low‐metabolic rate neighbor colonies on panels where flow was higher as they baffled the current more. We also found that the effects of the metabolic rate of neighbors differed in persistence and sign across our replicated panels—we suspect this variable effect arises because of small scale differences in currents. On higher flow panels, focal colonies may have benefited from low metabolism, large neighbors baffling flows, but on low flow panels, focal colonies suffered in the presence of such baffling (Svanfeldt et al., 2017).

We only investigated pairwise interactions between conspecifics, but intraspecific interactions occur across a range of densities in nature. Population density has been shown to affect the mode of competition (Cameron et al., 2007) as well as mediate transitions between competition and facilitation among species at least (Cameron et al., 2019). Similarly, the frequency of a given metabolic phenotype within a population may alter the outcome of interactions among conspecifics (Ayala & Campbell, 1974). Therefore, an important next step would be to orthogonally manipulate both the density *and* frequency of individuals of known metabolic phenotypes within a population and test for facilitative and competitive interactions.

Among species, context‐dependent changes in the strength of competitive interactions are an important maintainer of species coexistence (Chesson, 2000a, 2000b; Hart & Marshall, 2013). Similarly, it would be reasonable to expect that spatial variability in strength and direction of conspecific interactions maintains within‐population variation in metabolic rate (Pettersen et al., 2020). Although we found differences in conspecific interactions among microenvironments, the metabolic phenotype of focal colonies covaried with growth but had little effects on their survival or reproductive fitness. Specifically, we found that the focal metabolic rate affected the survival of focal colonies on one panel (interacting with the metabolic rate of the neighboring colony), but we could not detect an effect of the focal metabolic rate on either survival or reproductive outputs on the other panels. Instead, the performance of focal individuals on these panels was more consistently affected by the metabolic phenotype of their neighbor. Neighbor metabolic rate affected the survival, growth, and reproduction of focal colonies, albeit in contrasting ways that varied among microenvironments. These results suggest that complex eco‐evolutionary feedbacks (akin to “indirect genetic effects,” sensu Wolf et al., 1998) are likely to maintain variation in metabolic rate despite previous studies showing strong directional selection (that should erode phenotypic variation) on focal metabolic rate in this system (Pettersen et al., 2020). An important next step will be to determine the degree to which metabolic rate is heritable in this system.

That the metabolic rate of the neighbor colony had more pervasive effects on focal colonies than did the metabolic rate of those colonies themselves surprised us. Most studies to date have focused on covariance between the focal organism's metabolism and the performance of that organism (Pettersen et al., 2018). We can find few examples of studies that explore how the metabolic rate of one individual affects the performance of other individuals (Auer et al., 2020). Yet it is well understood that metabolic rate covaries with any number of traits that determine how an organism will interact with and affect its environment and other species (e.g., body size, foraging rate, resource use; Biro & Stamps, 2010; Cameron et al., 2019; Careau et al., 2008). Thus, in this context, perhaps our results are less surprising than they first appear.

We recommend that future studies of the ecological effects of metabolism expand their scope to investigate competitive interactions both within and among species and where possible, be done under field conditions. We predict that variation in metabolic rate is likely to have effects that extend beyond the focal organism but for the most part, these effects are unexplored. Our study highlights the importance of replicating arrays of competitors under natural conditions. We replicated our response surface design in space and found very different effects from one microsite to another—in the absence of such replication, we would have overestimated the consistency of metabolic effects and drawn potentially misleading conclusions about how metabolism affects competitive interactions. Instead, we found that, while the metabolic phenotypes of both focal individuals and their neighbors matter, their effects can differ in strength and direction—capturing this variability is necessary for a more complete understanding of such effects in nature. Future studies are necessary to determine why we see such variable effects of metabolic rate among microenvironments, but we suspect a small‐scale variation in current regimes and the delivery of resources—future studies will manipulate local food availability to determine its role (Svensson & Marshall, 2015).

## CONFLICT OF INTEREST

The authors declare no conflict of interest.

## AUTHOR CONTRIBUTIONS

**Lukas Schuster:** Conceptualization (equal); investigation (lead); methodology (equal); project administration (lead); validation (lead); visualization (lead); writing–original draft (lead); writing–review and editing (equal). **Craig R. White:** Conceptualization (equal); supervision (supporting); writing–review and editing (equal). **Dustin J. Marshall:** Conceptualization (equal); formal analysis (supporting); supervision (lead); writing–review and editing (equal).

## Data Availability

All data presented in this study are available at Dryad (https://doi.org/10.5061/dryad.xksn02vh7).
